# Tim17 Updates: A Comprehensive Review of an Ancient Mitochondrial Protein Translocator

**DOI:** 10.3390/biom10121643

**Published:** 2020-12-07

**Authors:** Minu Chaudhuri, Chauncey Darden, Fidel Soto Gonzalez, Ujjal K. Singha, Linda Quinones, Anuj Tripathi

**Affiliations:** Department of Microbiology, Immunology, and Physiology, Meharry Medical College, 1005 Dr. D.B. Todd, Jr., Blvd, Nashville, TN 37208, USA; cdarden17@mmc.edu (C.D.); fgonzalez18@mmc.edu (F.S.G.); usingha@mmc.edu (U.K.S.); lquinones17@mmc.edu (L.Q.); atripathi@mmc.edu (A.T.)

**Keywords:** mitochondria, protein import, TOM, TIM, trypanosomes, Tim17

## Abstract

The translocases of the mitochondrial outer and inner membranes, the TOM and TIMs, import hundreds of nucleus-encoded proteins into mitochondria. TOM and TIMs are multi-subunit protein complexes that work in cooperation with other complexes to import proteins in different sub-mitochondrial destinations. The overall architecture of these protein complexes is conserved among yeast/fungi, animals, and plants. Recent studies have revealed unique characteristics of this machinery, particularly in the eukaryotic supergroup Excavata. Despite multiple differences, homologues of Tim17, an essential component of one of the TIM complexes and a member of the Tim17/Tim22/Tim23 family, have been found in all eukaryotes. Here, we review the structure and function of Tim17 and Tim17-containing protein complexes in different eukaryotes, and then compare them to the single homologue of this protein found in *Trypanosoma brucei*, a unicellular parasitic protozoan.

## 1. Introduction

Mitochondria, the essential organelles in eukaryotes, perform various cellular functions. In addition to producing 80% of cellular ATP, mitochondria are also involved in calcium homeostasis, innate immunity, regulation of reactive oxygen species, critical metabolic pathways, and apoptosis [1,2,3]. Classical mitochondria possess two aqueous compartments enclosed by two membranes. The outermost membrane is the mitochondrial outer membrane (MOM), which uses multiple pores formed by voltage-dependent anion channels (VDAC) that allow small molecules such as metabolites and ions to pass through [4]. The second membrane is the mitochondrial inner membrane (MIM) that surrounds the innermost matrix compartment and is folded to form cristae. MIM is impermeable even to ions but studded with many membrane integral transporters and enzymes responsible primarily for cellular energy production [5,6]. The aqueous intermembrane space (IMS) separates the two membranes, except near the contact sites, where MOM and MIM are in close proximity [7]. At the center, the aqueous matrix houses the remnant genome of the eubacterial endosymbiont, which encodes only 1% of proteins required for the organellar function. Most of the mitochondrial proteins are coded in the host nuclear genome. These proteins are translated on cytosolic ribosomes and imported into various sub-mitochondrial locations via different receptor/translocase complexes in the MOM, MIM, and IMS [8,9]. The import of nucleus-encoded proteins into mitochondria is a conserved phenomenon among eukaryotes. The mitochondrial remnants, known as mitosomes and hydrogenosomes, found in some unicellular anerobic eukaryotes, including *Entamoeba*, *Giardia*, and *Trichomonads* species, respectively, also import proteins from the cytosol to perform their functions [10].

## 2. Mitochondrial Protein Import Machinery

Mitochondrial protein import machinery has been studied mostly in the eukaryotic supergroup Opisthokonta, which includes fungi/yeast and mammals, and in Archaeplastida (plants). The general concept developed from these elaborate studies indicates that nuclear-encoded mitochondrial proteins are translocated via specific protein complexes in the MOM and MIM, known as the translocase of the mitochondrial outer and inner membranes (TOM and TIM, respectively). Proteins that are destined to the mitochondria are discriminated from other cytosolic proteins by the presence of mitochondrial targeting signals (MTSs) [11,12]. The following three major types of MTSs exist: (1) a cleavable N-terminal, (2) a cleavable N-terminal along with a sorting signal, and (3) an internal type (Table 1). Another type of signal is found either at the N- or C-terminus of certain MOM proteins, known as the signal and tail-anchored proteins, respectively [13] (Table 1). For a few MIM proteins, an internal presequence-like segment followed by a hydrophobic patch also acts as the targeting signal [14,15] (Table 1). The import pathway of a nuclear-encoded protein depends on the type of targeting signal it has and also on its final destination in the mitochondria [12,16,17].

**Figure 1:**
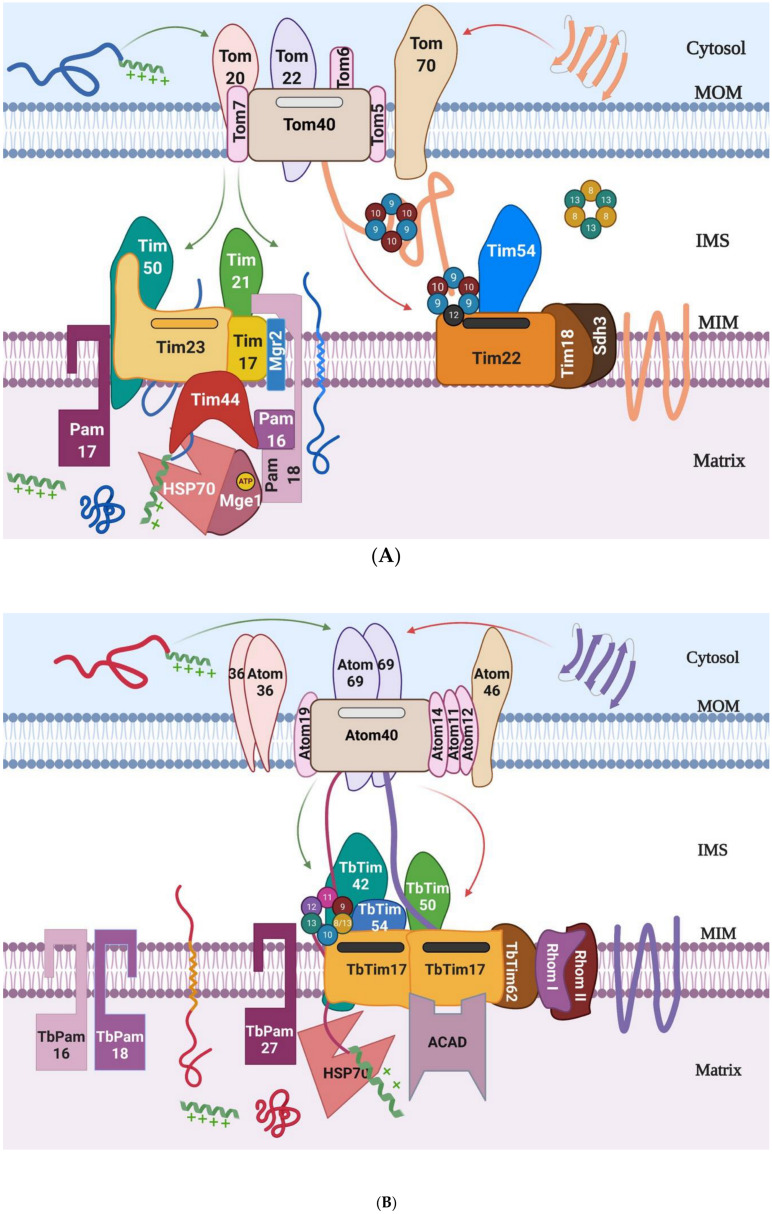
(**A**) The translocases of the mitochondrial outer and inner membranes, TOMs and TIMs, in *Saccharomyces cerevisiae*. TOM complex consists of Tom40, Tom70, Tom20, Tom22, Tom5, Tom6, and Tom7. The two TIM complexes are TIM23 and TIM22. The core components of the TIM23 complex are Tim23, Tim17, and Tim50. Tim21 helps to connect TIM23 with the TOM complex and often with the respiratory complex III. Mgr2 couples Tim21 to TIM23 core. TIM23 associates with the PAM complex that consists of Tim44, Hsp70, Pam16, Pam17, Pam18, and MgeI. The major component of the TIM22 complex is Tim22, and other components include Tim54, Tim12, Tim18, and Sdh3. The five small TIMS are Tim8, Tim9, Tim10, Tim12, and Tim13. The Tim9, Tim10 and the Tim8, Tim13 form two separate heterohexameic complexes in the intermembrane space that carry cargo proteins from the TOM complex to TIM22 complex. (**B)** The ATOM and the single TIM complex in *Trypanosoma brucei*. The major component of the ATOM complex, Atom40, and other subunits Atom14, Atom11, Atom12, Atom19, Atom46, and Atom69 are shown. Atom36 often associates with Atom40 but is not a part of this complex. The major component of the TbTIM complex is TbTim17. The TbTim62, TbTim42, Rhom I, and Rhom II are membrane integral proteins. TbTim50 is also membrane integrated but possesses a C-terminal domain exposed in the IMS. TbTim54 is a peripherally associated IMS protein. Location of ACAD is not known but expected to be in the matrix. Recent studies have shown that TbPam27 was membrane bound. Small TbTims are associated with the TbTim17 complex. The Cytosol, mitochondrial outer membrane (MOM), Intermembrane space (IMS), mitochondrial inner membrane (MIM), and matrix are labeled. The picture was drawn using the program (Biorender) available online.

The TOM complex virtually imports all nuclear-encoded mitochondrial proteins [24]. The major component of the TOM complex is Tom40, a beta(β)-barrel protein that forms a channel for protein translocation [25,26]. Additional components, i.e., Tom20, Tom70, Tom22, and Tom5 are important for translocase receptor function (Figure 1A). The N-terminal MTS-containing proteins are generally recognized by Tom20, and then are transferred to Tom22 before entering into the Tom40 channel, whereas Tom70 acts as the receptor for proteins with multiple internal targeting signals. Tom5 plays a role in the import of several IMS proteins. Tom6, Tom7, and Tom22 are involved in the structural assembly of the complex. Recent cryo-electron microscopy (cryo-EM) structure indicates that each Tom40 pore is surrounded by small Toms and tethered by two Tom22 subunits [27]. The homologues of most of the Tom subunits have been identified and characterized from mammals and plants [28,29]. However, a homologue of Tom70 appears absent in plants, instead a plant-specific protein Om64 (which is a paralogue of the chloroplast outer membrane import receptor, Toc64) functions in a similar manner [29,30]. In addition to Tom40, MOM also possesses a few other β-barrel proteins, which are inserted into the MOM by topogenesis of the β-barrel protein (TOB) complex, also named as the sorting and assembly machinery (SAM) [31]. The major component of this complex is Tob55/Sam50, a well conserved β-barrel protein. In addition to β-barrel proteins, MOM has several α-helical transmembrane proteins. Most of these proteins are translocated via the mitochondrial import (MIM) complex [13]. Tom40 homologues have also been found in mitosomes and hydogenosomes [32,33,34,35]. Mitosomes are an extremely reduced form of mitochondria found in certain anaerobic protists, such as *Giardia*, *Microsporidia*, *Cryptosporidium*, and *Entameoba*. Mitosomes do not generate ATP but perform a conserved function of mitochondria, i.e., biosynthesis of the Fe-S clusters [10]. Similarly, hydrogenosomes are also the relict form of the mitochondria found in *Trichomonad* species. Unlike mitosomes, hydrogenosomes produce ATP by substrate-level phosphorylation and generate hydrogen as a by-product [10]. These organelles are surrounded by two membranes but lack any genetic material and the cristae [32,34]. Therefore, all of these organellar proteins are imported from the cytosol.

After crossing the TOM complex, the N-terminal MTS-containing proteins, targeted to the mitochondrial matrix, are translocated via the TIM23 complex that also contains the presequence translocase-associated motor (PAM) components [18,36] (Figure 1A). After entering the matrix, the presequence is cleaved by the mitochondrial processing peptidase (MPP). Many inner membrane proteins that have a sorting signal along with an N-terminal MTS are also translocated via the TIM23 complex. Once the sorting signal enters the TIM23 channel, translocation halts, the motor subunits dissociate, and preproteins are laterally sorted into the MIM [37,38]. Additionally, a large group of MIM proteins that have multiple transmembrane domains, such as mitochondrial carrier proteins (MCPs), are recognized by their internal targeting signals and translocated by the TIM22 complex [39,40,41].

Similar to the TOM, these TIMs are multi-protein complexes. The core components of the TIM23 complex in yeast are Tim23, Tim17, and Tim50, whereas those of the TIM22 complex in yeast/fungi are Tim22, Tim54, Sdh3, and Tim18 [12,36,41] (Table 2). Tim23, Tim17, and Tim22 belong to the same protein family [42]. Tim23 and Tim22 are the channel-forming units of the TIM23 and TIM22 complexes, respectively [43,44], whereas Tim17 acts as the structural component of the TIM23 complex and also plays a regulatory role for the Tim23 translocase activity [45]. Tim50 acts as the receptor for the TIM23 complex [46,47] (Figure 1). The N-terminal targeting signal, or the presequence that emerges from the trans-site of the TOM complex, is recognized by Tim50 for further translocation through the Tim23 channel [46,47]. Tim21 and Mgr2 associates with the TIM23 complex during translocation of the MIM protein with a sorting signal [48]. The components of the TIM22 complex are less conserved relative to the components of the TIM23 complex between fungi and humans [49]. Among the core components of the TIM22 complex, Tim54 and Tim18 are only found in the yeast TIM22 complex and these proteins are required for stability of this translocase [22,50]. No homologues of Tim54 and Tim18 exist in humans. Instead, a novel protein, Tim29, is found to be critical for the structure and function of the human TIM22 complex [21,49] (Table 2). Acylglycerol kinase (AGK) is also a component of the human TIM22 complex [51]. Recently, the cryo-EM structures of both the human and yeast TIM22 complex have been reported [52,53]. Analysis of the yeast TIM22 structure raises the question if Tim22 forms a twin-pore structure, as reported earlier, or the single Tim22 subunit forms a TM insertase-like bacterial YidC [53]. It is likely that yeast TIM22 at the resting stage forms the insertase-like structure, however, upon activation by membrane potential, could dimerize to form the twin-pore conformation [53].

The PAM complex in yeast also has multiple components (Figure 1A). Tim44, a scaffold protein, is peripherally associated with MIM and exposed in the matrix. Tim44 recruits heat shock protein 70 (Hsp70) associated with a nucleotide exchange factor, Mge1, and also connects the TIM23 complex with the PAM complex [54,55]. Other PAM components include the J domain-containing protein, Pam18/Tim14, and J-like proteins, Pam16/Tim16 and Pam17 [55,56] (Table 2). These Pam components also make direct contact with Tim17 and Tim23 and regulate protein translocation. Similar Pam proteins have been found in plants and mammalian systems [29,57] (Table 2). Human DnaJC19 and DnaJC15 are the homologues for Pam18 of yeast. Likewise, the Pam16 homologue is referred to as Magma and mitochondrial Hsp70 is called Mortalin or HSPA9 in humans [57]. The consensus is that mitochondrial membrane potential and ATP hydrolysis are prerequisites for translocation of proteins through the MIM to the matrix [37]. Mitochondrial membrane potential is required for activation of the TIM23 translocase and for electrophoretic movement of preproteins towards the matrix. The internal signal-containing proteins also require mitochondrial membrane potential for their insertion by the TIM22 complex [44].

Internal signal containing MIM proteins enter into the TOM channel as a loop structure and are assisted by the small Tim chaperones [58]. In yeast, the hetero-hexameric complex formed by Tim9 and Tim10 (3:3) in the IMS is primarily used for the release of these hydrophobic proteins from the TOM complex for translocation to the TIM22 complex [59] (Figure 1A). Then, the cargo proteins are further transferred to a Tim9-Tim10-Tim12 (3:2:1) complex located on the TIM22 translocase for translocation [60,61]. Tim12 is stably associated with Tim22. Tim8 and Tim13 also form a soluble hetero-hexameric complex similar to Tim9-Tim10. However, Tim8 and Tim13 are not essential under normal conditions in yeast. Some of these small Tim chaperones also assist MOM β-barrel proteins to be translocated to the SAM complex for assembly [62]. After entry into the IMS through the TOM complex, small Tims are oxidatively folded by the mitochondrial import and assembly (MIA) complex [63]. Humans also possess multiple small Tim proteins, i.e., Tim9, Tim10a, Tim10b, and DDP1 (Tim8). Tim9 and Tim10a form a complex, whereas Tim10b performs functions similar to Tim12 of yeast [64]. In addition to fungi and mammals that belong to the eukaryotic supergroup Opisthokonta, the Tom and Tim components vary widely in number, size, and homology among other eukaryotic groups. The most remarkable divergence has been found in excavates, which include *Trypanosoma* and *Leishmania*, that diverge very early during evolution [65,66].

Recently, mitochondrial protein import machinery in *T. brucei* has emerged from the studies by groups of investigators. An elegant work from Schneider’s group identified and characterized the homologue of Tom40 in *T. brucei* and named this protein as archaic Tom40 or Atom40 [67]. Reconstitution of the recombinant Atom40 on lipid bilayer showed that the protein could form a cation-sensitive channel, which was more similar to the channel of the bacterial protein Omp85 than eukaryotic Tom40 [68]. Atom40 associates with several other trypanosome-specific proteins, such as Atom11, Atom12, Atom14, Atom19, Atom46, and Atom69 [69] (Figure 1B). It is thought that that Atom69 and Atom46 act as the receptor for incoming polypeptides, similar to Tom70 and Tom20 in yeast/fungi, however their substrate specificities could be different [68]. Atom14 is distantly related to Tom22 in other eukaryotes [70]. *T. brucei* also possesses a TIM complex. The major component of this complex is TbTim17 which was initially found by homology searches after both the *T. brucei* genome sequence and mitochondrial proteomes were completed (www.tritrypDB.org). We showed that TbTim17 was essential and involved in mitochondrial protein import in two major developmental stages of *T. brucei* [71]. *T. brucei* does not have any additional homologues for Tim23 or Tim22. However, TbTim17 is present in larger size protein complexes within the range of 300 kDa to 1100 kDa [72,73]. Further work has revealed that TbTim17 was associated with multiple trypanosome-specific proteins. These included TbTim62, TbTim42, TbTim54, TbTim50, Rhomboid-like proteins I and II, acyl-CoA dehydrogenase (ACAD), and a group of small TbTims [72,74,75] (Figure 1B). Functions of each of these proteins and their stoichiometric ratio in the TbTIM17 complex need to be explored. *T. brucei* also possesses homologues of Pam16, Pam18, Mge1, and mitochondrial Hsp70. Tyc and colleagues showed that MgeI and Hsp70 were needed for maintenance and replication of mitochondrial DNA, also known as kinetoplast or kDNA [76,77], which could be additional functions of these proteins. In addition, *T. brucei* possesses a putative Tim44 homologue and TbPam27, which is found as a trypanosome-specific Pam component [78] (Figure 1B). Co-imminoprecipitation analysis has shown that TbTim17 was associated with mitochondrial Hsp70 [72,79]. Together, it suggests that *T. brucei* possesses a PAM complex.

In addition to mitochondrial protein import, *T. brucei* ATOM and TbTIM complexes are found to be involved in the import of tRNAs into mitochondria. Unlike in other eukaryotes, trypanosome mitochondrial DNAs do not encode any tRNAs, therefore, all tRNAs needed for mitochondrial translation are required to be imported into mitochondria [80]. Recent work from Schneider’s group showed that some of the ATOM components were directly involved in translocation of tRNA^Ile^ into mitochondria [81]. Chaudhuri’s lab showed that knockdown of TbTim17 and TbTim62 significantly reduced the levels of 16 and 13 tRNAs into mitochondria, respectively [82], indicating the role of these protein translocases in the import of tRNAs into *T. brucei* mitochondrion. However, all components of these complexes are not required for this process, suggesting that smaller modular complexes with few common translocator proteins are sufficient for tRNA import.

Trypanosomatids possess both N-terminal and internal targeting signal-containing, nucleus-encoded mitochondrial preproteins [15,83]. Furthermore, these types of preproteins, as well as the MOM β-barrel protein from heterologous sources, are capable of being imported into trypanosomatid mitochondria both in vitro and in vivo [73,84]. Recent studies have shown that both the N-terminal and internal targeting signal-containing substrate proteins were associated with many common subunits of the TbTIM17 complex while arrested during translocation. This suggests that *T. brucei* most likely possesses a single TIM complex [75]. TbTim17 and TbTim62 are also found to be involved in the import of tRNAs into *T. brucei* mitochondrion [82,85]. How TbTim17 functions to import such a diverse group of substrates remains to be elucidated. It can only be speculated that TbTim17 forms modular sub-complexes by association with certain trypanosome-specific proteins to perform such diverse jobs. Therefore, it is highly significant to study these divergent organisms to understand how different ways the translocators may work.

## 3. Prevalence of Tim17 and Tim17 Homologues Among Eukaryotes

Tim17 and the Tim17/22/23 family proteins have been found in all eukaryotes. The origin of this group of proteins is controversial. Initially, it was believed that the Tim17/22/23 proteins belong to the bacterial AA transporter family, such as LivH, due to the presence of a signature motif known as the presequence and AA transporter (PRAT), ((G/A)X_2_(F/Y)X_10_RX_3_DX_6_(G/A/S)GX_3_G) [42]. However, further analysis revealed that besides the presence of the glycine-zipper motif (GX_3_GX_3_G), very little evidence proved that LivH was the prokaryotic ancestor of the Tim17/22/23 family proteins in eukaryotes [86]. Mitochondrial Mgr2 in yeast and its human homologue Romo1 also belong to the Tim17 family [86]. Additionally, members of this protein family have been found in organelles other than mitochondria, such as plastids like HP20, HP30 [87], and peroxisomes, i.e., Pmp24 and Tmem135 [88]. In addition to the mitochondrial protein translocases, members of this family have also been found in other protein complexes in the mitochondria, such as the NADH dehydrogenase complex (Complex-I) [89,90]. Although common in fungi, animals, and plants, the presence of the three homologous mitochondrial translocase proteins, Tim17, Tim22, and Tim23, vary in the number of copies among different species (Table 3). Humans have two copies each for Tim17 and Tim23 [16] and studies on the model plant Arabidopsis have identified three copies for each of these proteins [29]. Interestingly, trypanosomatids possess only the single member of this family protein, which is TbTim17 [91]. The primary sequence of TbTim17 have shown 29.7%, 31.2%, and 21.8% similarity and 18%, 16.8%, and 7.1% identity with the primary sequence of ScTim17, ScTim22, and ScTim23, respectively, suggesting that TbTim17 had closer similarity either to ScTim17 or ScTim22 than to ScTim23 [73]. Although some confusion exists as to whether trypanosomatid Tim17 is a true homologue of the canonical Tim17 or Tim22 of fungi/yeast, we showed that ScTim17 could complement partially the growth defect of *T. brucei* caused by TbTim17 RNAi, as ScTim17 was targeted properly into mitochondria and assembled into the TbTim17 protein complex, whereas ScTim23 was imported into *T. brucei* mitochondria but not associated with TbTim17. Conversely, ScTim22 was not imported into *T. brucei* mitochondria. Therefore, neither ScTim23 nor ScTim22 could complement the growth defect of *T. brucei* caused by TbTim17 knockdown [73]. These results indicate some functional similarity of TbTim17 with ScTim17. However, TbTim17 did not complement the *S. cerevisiae* mutants of either ScTim17, ScTim22, or ScTim23, despite of the fact that TbTim17 was expressed and localized in yeast mitochondria [73], suggesting the incompatibility of TbTim17 in yeast mitochondrial protein import machinery. There is a conception that TbTim17 could be functionally similar to ScTim22, as TbTim17 has been shown to be involved in the import of internal signal-containing proteins in addition to the N-terminal preproteins into mitochondria [75], however this concept warrants further investigation. Unlike trypanosomatids, other members of the eukaryotic supergroup Excavata, including *Trichomonas vaginalis* (*T. vaginalis*) and *Naegleria gruberi*, possess multiple copies of the Tim17/22/23 family proteins [32,34] (Table 3). Recently, a Tim17-like protein also has been identified in *Giardia* mitosomes [92]. The *Giardia* Tim17 showed a stronger similarity to Tim17 than Tim23 and to Tim22 in other eukaryotes [34]. *T. vaginalis* possesses two proteins (TvTim17/22/23-1 and TvTim17/22/23-2) with stronger similarity to the Tim17/22/23 family and to three other proteins with lesser similarity [Rada et al. 2011]. The former two isoforms are speculated to be the part of the TIM complex in *T. vaginalis* hydrogenosomes. Among these, TvTim17/22/23-1 possesses special features of Tim23, such as the positively charged residues at the C-terminus and negatively charged residues at its N-terminus. However, due to low conservation of these hydrogenosomal proteins, it was difficult to group them properly with the Tim17, Tim22, or Tim23 subfamilies. Furthermore, neither of the TvTim17/22/23-1 and TvTim17/22/23-2 could complement growth of the yeast strain lacking Tim17, Tim22, or Tim23. Additionally, the existence of the triad members, Tim17, Tim22, and Tim23, have been found in the last eukaryotic common ancestor [86]. Thus, the presence of a single Tim17 family protein in trypanosomatids and *Giardia* is likely the secondary loss during their divergent evolution.

## 4. Tim17 and Tim17-Containing Protein Complexes in Different Eukaryotes

In the early 1990s, Tim17/MIM17 was first identified in yeast as an essential protein that could complement the mitochondrial protein import defect [98]. Later, it was shown that Tim17/MIM17, along with another homologous protein, Tim23/MIM23, formed a complex of 280 kDa [99]. This membrane integral complex associates with Tim44, a peripheral MIM protein, and Hsp70 to form the TIM23 complex [100], which is often associated with components of the TOM complex, particularly during translocation of proteins. Tim23 and Tim17 (without any other proteins) form a 90 kDa complex with two copies each of these subunits [101]. There are some evidences that Tim23 forms two distinct complexes in yeast as follows: (1) TIM23motor and (2) TIM23sort [102]. The TIM23 motor complex is formed by association of the TIM23 core with the PAM complex and translocates matrix-targeted preproteins, whereas, in TIM23sort, the core TIM23 complex does associate with Tim21 but not with the PAM complex and is involved in translocation of the sorting signal-containing preproteins [103].

The homologues for Tim17 in humans were cloned and characterized in 1999 [16]. Two copies of Tim17 genes, hTim17a and hTim17b (Table 3), are localized in the short arm of the X-chromosomes. The human Tim17b (hTim17b) protein exists in two isomeric forms, hTim17b1 and hTim17b2. In association with hTim23 and other proteins, hTim17a and hTim17b form two distinct complexes, translocase-A and -B [94]. Translocase-B is constitutive and essential for normal mitochondrial functions, and translocase-A is non-essential under normal conditions but essential for oncogenic cell proliferation. In plants, three copies of Tim17 have shown tissue-specific expression [29]. However, it was unclear whether they formed distinct sub-complexes along with multiple Tim23 proteins in plants.

In *T. brucei*, TbTim17 is found in various-sized protein complexes within the range of 300 kDa to 1100 kDa, along with some trypanosome-specific Tims [72,75]. By tandem affinity purification (TAP) of the TbTim17 protein complex, the Chaudhuri lab identified at least two novel components, TbTim62 and TbTim54 [72] (Table 2). We showed that TbTim62 is a MIM protein and was required for stability of the TbTim17 protein complex [79], whereas TbTim54 is peripherally associated with the MIM and exposed in the IMS and is involved in the import of internal signal containing proteins including TbTim17 [104]. We also identified a homologue of Tim50 and verified that it was associated with TbTim17 [74]. Using reciprocal co-immunoprecipitation and SILAC proteomics, the Schneider’s group identified additional components of the TbTim17 complex, such as TbTim42, two rhomboid-like proteins, and several small TbTims [75] (Table 2). ACAD, a metabolic enzyme, has been consistently precipitated by the TAP-tag approach and by quantitative immunoprecipitation analysis [72,75]. However, further investigation is required to verify its role in mitochondrial protein import. Interestingly, TbTim50 was not identified by proteomics analysis of the purified proteins, either by TAP or co-immunoprecipitation analyses [72,75], and that could be due to its weak association with TbTim17 or to other technical errors. Conversely, TbTim54 has been identified only by the TAP-tag approach [72]. There are several small Tim homologues in trypanosomatids [66,105,106]. *T. brucei* possesses at least six small Tims, TbTim9, TbTim10, TbTim11, TbTim12, TbTim13, and TbTim8/13 [105,106]. Except for TbTim12, all of these small TbTims possess a pair of characteristic CX3C motifs. TbTim12 possesses two imperfect motifs, where one C in each motif is missing. TbTim8/13 shows homology to bothTim8 and Tim13 in yeast. All of these small TbTims are essential for *T. brucei* cell growth and found tightly associated with TbTim17. Depletion of any of these severely hampers the stability of the TbTIM17 complex [66,105,106], which appears unique for this organism.

## 5. Tim17 Structure

**Figure 2:**
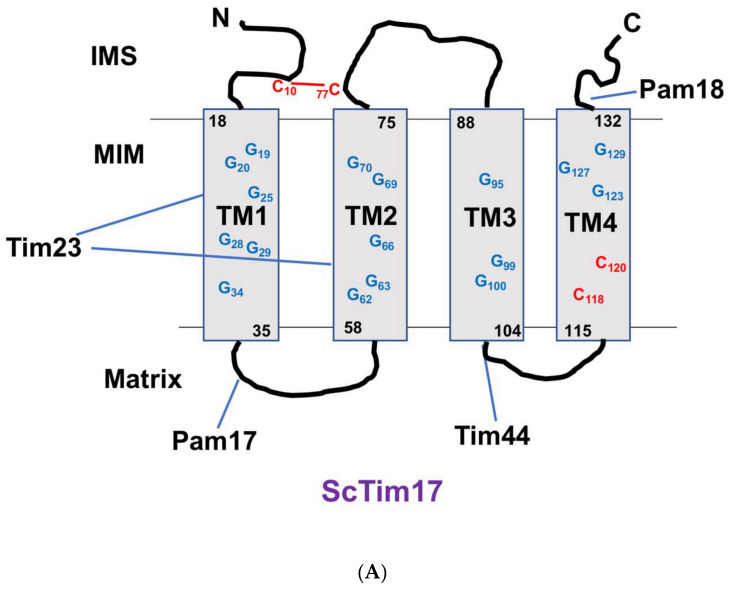

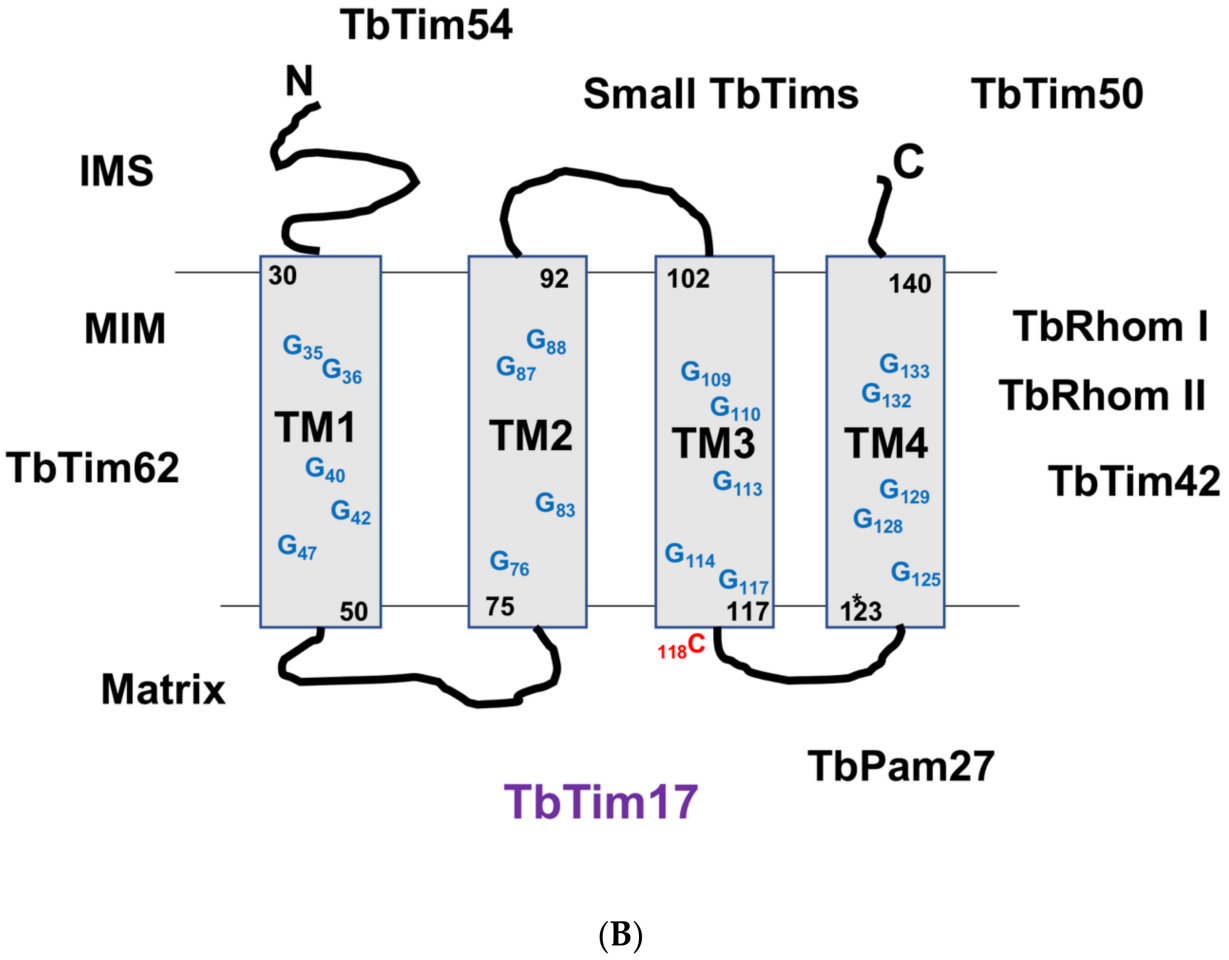
Schematics of the secondary structures of Tim17 of the *Saccharomyces cerevisiae* (**A**) and *Trypanosoma brucei* (**B**). Four transmembrane domains (TM1–TM4) and the hydrophilic loop regions are shown. Glycine (G in blue) residues within the TMs and cysteine (C in red) residues are indicated. Numbers indicate the position of these Gs and Cs in ScTim17 and TbTim17 sequences. Cs that form the disulfide bond in ScTim17 are connected with lines. IMS, intermembrane space; MIM, mitochondrial inner membrane; and the matrix are indicated. Interacting regions of ScTim17 with Tim23, Pam17, Pam18, and Tim44 are indicated. Interacting regions of TbTim17 with other subunits are yet to be determined. However, the known localization of these subunits in the IMS, MIM, and matrix is shown.

Tim17 has four transmembrane domains (TMs), and the N- and C-termini are in the IMS. This topology has been found conserved for the Tim17/22/23 family proteins. Although some exceptional structures exist in certain members, the ScTim23 has N-terminal extension of 100 AA residues [107]. This region is responsible for Tim23 dimer formation and interaction with the IMS-exposed regions of Tim50, Tim21, and Tom22 [47,108,109]. It has been shown that the N-terminal of ScTim23 crosses the MOM to facilitate the connection between the TOM and TIM23 complex [110]. Furthermore, this double membrane-spanning conformation of the ScTim23 N-terminal is flexible and dependent on the presence and absence of mitochondrial membrane potential [111,112]. In contrast to ScTim23, *Arabidopsis thaliana* Tim17-2 (AtTim17-2) possesses a C-terminal extension of 143 AA residues, which extend through the MOM to facilitate interaction with the TOM and also substrate proteins. Deletion of this C-terminal extension of AtTim17-2 enables this protein to complement the ScTim17 mutant, indicating a plant-specific structure of Tim17 required for its function in plant mitochndria [95,96]. The secondary structure prediction of TbTim17 showed four TMs, three loops, and two hydrophilic termini. The predicted positions of the TMs and the lengths of the loop regions of TbTim17 are more similar to those of ScTim23 and ScTim17 than to those of ScTim22 [73]. The N-terminal hydrophilic region of TbTim17 (1 to 29 amino acid residues) is much shorter than the corresponding region in ScTim23 and slightly longer than that seen in ScTim17. In contrast, the C-terminal hydrophilic region of TbTim17 is relatively longer than that seen in ScTim23 but shorter than that in ScTim17 [73]. Besides the similarity of its secondary structure, the primary sequence of TbTim17 is significantly divergent than Tim17 in yeast and human, particularly at the N-terminal domain. However, TbTim17 possesses the signature PRAT motif within TM2-loop2-TM3 region and multiple GXXXG motifs within the TMs, as discussed in the later section [73] (Figure 2). The most divergent Tim17 sequence has been found in *Giardia* (Gi), which was recognized by specialized Hidden Markov Model [92]. In spite of this divergence, GiTim17 has the conserved arginine residue for interaction with Tim44 and also has one GXXXG motif in one of the predicted TMs at the C-terminal domain [92].

## 6. Biogenesis of Tim17

Tim17 is encoded in the nuclear genome; therefore, it is required to be imported into the mitochondria and inserted into the MIM once synthesized in the cytosol. It has been shown that human (h), drosophila (d), and yeast Tim17s were imported in vitro into rat liver mitochondria [36]. The hTim17 and dTim17 also were inserted into the inner membrane and associated with ScTim23 in the yeast mitochondria in vitro suggesting that the Tim17 import pathway is conserved in these species. Tim17, Tim22, and Tim23 are very similar MIM proteins; yet some differences in their import pathway have been noted. Studies in model yeast have shown that ScTim17 requires the ScTom70 receptor for entering into the TOM complex [54,113]. The ScTim17 requires both TIM23 and TIM22 complexes for MIM insertion and assembly, but ScTim23 solely depends on TIM22 to reach its destination. In addition, Tim23 in yeast and humans depends on the Tim8-Tim13 complex for translocation through the IMS [114,115,116], whereas Tim17 likely depends on the Tim9-Tim10 chaperone complex to cross the IMS.

Tim17, Tim22, and Tim23 do not have the N-terminal mitochondrial targeting signal, and therefore depend on one or more internal targeting signals. Several attempts were made to identify these internal signals in yeast proteins. It has been shown that the first 50 AAs of ScTim23 are dispensable for the protein to be localized in the mitochondria and for being inserted into the MIM. However, the fusion protein consisting of the N-terminal 62 AAs of ScTim23 and dihydrofolate reductase (DHFR) [(1-62)Tim23-DHFR] was targeted to mitochondria [113]. The imported protein was protected from limited proteinase K digestion of mitochondria, but not when mitochondria were swelled before digestion suggesting that the fusion protein is localized in the IMS. This indicates that the 50–62 AA region could have some targeting signal but not for membrane insertion. TM domains likely are needed for the latter function. Based on the observations that 1 to 180 AAs of ScTim23 (that has the first two TMs) were targeted to mitochondria, but not inserted into the IMS, and ScTim23fs (C-terminal 16 AAs (TM3) were replaced by hydrophilic residue) is functional in vivo, it was indicative that TM4 is important for membrane insertion. Although in vitro studies revealed that ScTim23fs is imported but not inserted efficiently into the IM. Davis and colleagues reported that the positively charged residues of at least one out of two matrix-localized loops are needed for insertion of ScTim23 into the IM [115]. This group also showed that a pair of hydrophobic segments is required for efficient import of ScTim23 into mitochondria and TM4, in particular, is critical for this process. Overall, it suggests that ScTim23 is imported as a loop structure similar to MCPs and likely possesses more than one targeting signals. Similar studies for ScTim17 have revealed that neither the N-terminal 12 AAs nor the C-terminal 24 AAs are needed for targeting and insertion of this protein to the MIM or to be assembled with ScTim23, but this N-terminal region is critical for ScTim17 function [117]. However, deletion of the C-terminal 58 AAs, which removes the 4^th^ TM, hampered mitochondrial localization of ScTim17 [113]. Between TM3 and TM4 of both ScTim23 and ScTim17, there is a positively charged N-terminal MTS-like sequence. When fused with DHFR, this region is capable to target the fusion protein to the matrix [113]. However, the significance of this sequence for import and membrane insertion of ScTim17 and ScTim23 is unclear.

ScTim17 possesses four cystines: C10, C77, C118, and C120 (Figure 2A). Among these, C10 (located in the N-terminal hydrophilic region) and C77 (in the second loop) form an intramolecular disulfide bond, which is critical for its function but not for its membrane insertion [118,119] (Figure 2). The hTim17 has three Cs, and *Candida* (*Ca)* Tim17 has five Cs. All of these Tim17s have the same conserved intramolecular disulfide bond as ScTim17. As with Tim17, Tim22 also possesses multiple Cs, and the number varies among different species. ScTim22 has four Cs, CaTim22 has 5 Cs, and hTim22 has six Cs. In ScTim22, C40 (N-terminal hydrophilic region) and C141 (second loop) form an intramolecular disulfide bond, similar to that in Tim17. However, unlike Tim17, this S-S bond formation in ScTim22 is critical for membrane integration of this protein. Furthermore, ScTim22 membrane insertion depends on Mia40. In contrast to Tim17 and Tim22, Cs in Tim23 are not conserved, and are present in a reduced form.

A recent study from Chaudhuri-lab on *T. brucei* revealed that deletion of the N-terminal 30 AAs of TbTim17 did not hinder its targeting to mitochondria but largely hampered its assembly into the TbTIM complex [120]. These results suggest that TbTim17 possesses internal mitochondrial targeting signal(s) but the N-terminal region is possibly critical for interaction with other proteins necessary for its assembly. It has also been shown that TbTim17 expressed in yeast was able to be imported into mitochondria [73]. Similarly, ScTim17, ScTim23 expressed in *T. brucei* were localized into mitochondria, indicating that the mitochondrial targeting signal in TbTim17 was likely conserved with that in ScTim17. In contrast to ScTim17 and ScTim23, we found that ScTim22 was unable to be imported into mitochondria when expressed in *T. brucei* [73], revealing that a process required for ScTim22 import is absent in *T. brucei*. Since a homologue of Mia40 has not been found in *T. brucei*, it is conceivable why ScTim22 was not imported into *T. brucei* mitochondria. TbTim17 possesses a single cystine residue (C118), which is located at the very beginning of the third loop (Figure 2B). It is not clear, at this moment if this residue plays any role in intermolecular interaction or membrane insertion of TbTim17. Further studies are on-going to localize the targeting and membrane insertion signal(s) of TbTim17.

## 7. Tim17 Interaction with Other Proteins and Its Function in Mitochondrial Protein Import

In fungi, human, and plants, Tim17 interacts with Tim23 to form the core of the TIM23 complex. Mutagenesis analysis revealed that TM1 and TM2 of ScTim17 are responsible for ScTim17’s interaction with ScTim23 [121,122] (Figure 2A). ScTim17 also directly interacts with mHsp70 and weekly interacts with Tim44 [100]. Mutation of the AAs near the end of TM3 and in the third loop hampered the association of ScTim17 with the PAM complex [122]. Pam18 interacts with ScTim17 at the IMS site, whereas the first loop of ScTim17 interacts with Pam17 at the matrix site [55,56] (Figure 1). Electrophysiological studies have revealed that the TIM23 complex in yeast forms a twin-pore structure. Although, ScTim23 is the major channel-forming unit [8], depletion of ScTim17 collapses the twin pore to a single-pore conformation and also hampers the gating function of this complex [45].

ScTim17 possesses multiple GXXXG motifs within the TMs (Figure 2A). This motif is known to be involved with TM domain interaction among various proteins. Mutation of Gs in the TM1 (G25L, G29L), TM2 (G62L, G66L), and TM3 (G95L, G99L) of ScTim17 showed slow growth phenotype [122]. Among these mutants, G99L had a more adverse effect on growth and mitochondrial protein import. The G25L, G29L, G62L, and G66L Tim17 mutants could not form the 90 kDa core complex. Although G95L and G99L mutations did not hamper the formation of the 90K complex, the mutations disrupted the association of ScTim17 with Tim44. Out of the two matrix-exposed loops of ScTim17, L1, and L3, the first part of L3 (104–108 AAs) was found responsible for interaction of ScTim17 with the PAM components. ScTim17 R105A had loose interaction with Pam16 and Pam18 but not with Tim23. Cross-linking experiments revealed that ScTim17 R106 could be cross-linked with Tim44. Involvement of the TM1 and TM2 of Tim17 for its interaction with ScTim23 also was confirmed independently by another group [121]. This group made a series of single and double G-mutants, such as G25L/G29L in TM1; G62L/G66L, G62L/G70L, G66L/G70L in TM2; G95L, G99L in TM3; and G123L/G127L in TM4 and showed that all of these mutants have temperature-sensitive phenotypes and have reduced stability of the ScTim17. The order of ScTim17 stability among mutants was found as follows: WT > G62L/G70L > G62L/G66L > G66L/G70L > G123L/G127L > G95L/G99L > G25L/G29L > G99L/G107A > G99L/G107L. Accumulation of the Hsp60 precursor protein indicated that the import of mitochondrial proteins was hampered in G19A, G19A/A23L, A58L/G70L, G66L, G99L, and G123L/G127L mutants. Interaction with ScTim23 was hampered due to mutation of G62L/G66L, G62L/G70L, and G66L/G70L in the TM2. In addition to mitochondrial protein import defects, mutation of these G residues caused reduced membrane potential, increased mito-ROS, mitochondrial DNA loss, and mitochondrial fragmentation, indicating that Gs in each TM are critical for ScTim17 function. Tim17 in other species also possesses similar GX3G motifs in the TMs. TbTim17 has 19 Gs in four TMs (Figure 2B); however, the role of these residues, in interaction with other trypanosome-specific Tims, has not been determined yet.

The N-terminal hydrophilic region of ScTim17 is not critical for the stability or integrity of the TIM23 complex; however, truncation of this region showed a dominant negative effect on the mitochondrial membrane potential. In this region, two aspartate residues are present, which form salt bridges with two basic residues located in the L2, thus, creating a presequence-sensitive gate of the import channel [117].

In addition to the interaction with the Tim23 and Pam components, Tim17 also plays a role in connecting the TIM23 complex with the mitochondrial respiratory complex III in yeast and plants [123]. ScTim21 is a non-essential subunit of the TIM23 complex [124]. The single transmembrane domain of ScTim21 is anchored to the MIM, and the rest of the protein is in the IMS. The IMS region of ScTim21 interacts with the trans site of the TOM complex and facilitates contact-site formation between the MOM and MIM. The ScTim21-containing TIM23 complex interacts with the respiratory complex III and helps insertion of the sorting sequence containing MIM proteins [103,125]. Upon binding of ScTim17 with Pam18, ScTim21 dissociates, and TIM23 connects with the PAM complex for translocation of the matrix-targeted proteins.

## 8. Non-Canonical Functions of Tim17

### 8.1. Role of Tim17 in Mitochondrial Stress Response

Previously, it was thought that the import of proteins into mitochondria is a constitutive process. However, studies increasingly indicate that this process is often adapted according to the physiology of the mitochondria and cell by changing the levels of expression of specific Tims [126] and also by phosphorylation of specific TOM proteins [127,128]. Mutation of mitochondrial DNA during ageing or disease conditions, inhibition of mitochondrial translation, defect in mitochondrial protein import, and changes in the nutritional environments of cell, could cause a damage in the oxidative phosphorylation (OXPHOS) and increase ROS production. As a consequence, unassembled, mis-folded or aggregated proteins are accumulated. Increased ROS may also generate more damage in the mitochondrial genome, thus, amplified the process. Accumulation of mis-folded or aggregated proteins in the mitochondrial matrix induce mitochondrial unfolded protein response (mtUPR) that increases production of mitochondrial chaperones and proteases by retrograde signaling [129,130]. The mechanism of mtUPR has been studied in *Caenorhabditis elegans* (*C. elegans*) and in human cells. It has been shown that the levels of Tim17 and Tim23 play critical roles to elicit mtUPR response [126,131,132]. In *C. elegans*, misfolded mitochondrial proteins are cleaved by mitochondrial proteases, and generated peptides are exported by the Haf-1 protein. These exported peptides block the TIM23 channel and attenuate mitochondrial import of ATFS-1, a bZip transcription factor that possesses both MTS and nuclear localization signals. Under normal physiological conditions, ATFS-1 enters into mitochondria and rapidly degrades. However, when mitochondrial import is inhibited, ATFS-1 accumulates in the nucleus and triggers transcription of the stress-response genes, including mitochondrial chaperones, proteases, and transporters. Attenuation of mitochondrial protein import by reduction of the levels of either Tim23 or Tim17 by shRNA also elicits a similar response. In the mammalian system, Tim17A is known as a stress-regulated subunit of the TIM23 complex. Under stress, mitochondrial protease YME1L degrades Tim17A and its level is reduced, that creat mtUPR to stabilize mitochondrial homeostasis [126]. If the response is not sufficient to manage the deficit, defective mitochondria are destroyed by mitophagy. Mitochondrial protein import defect under stress also causes an accumulation of precursor proteins in the cytosol, which induces a stress response. Mix23 in yeast and its homologue CCDC58 in human are IMS proteins. It has been shown that Mix23 is up-regulated under stress and stabilizes ScTIM23 complex [133]. Several Pam components also have found to be critical to reduce ROS generated under mitochondrial stress. The nucleotide-exchange factor for Hsp70 of the hTIM23 complex, Mge1, plays an important role in scavenging mitochondrial ROS and protects cells from apoptosis upon external oxidative stress [134]. The ROS-scavenging property of human Magma is also evident. Although the mechanism is not clear, it has been shown that overexpression of Magma increased mitochondrial DNA stability and copy numbers that might increase the production of respiratory complex proteins and reduced ROS production [135]. In plants, Tim17-1 expression is increased in germinating seeds in response to salinity-induced stress response [136]. We found that overexpression of an ectopic copy of TbTim17 in *T. brucei* reduced the levels of the endogenous protein, indicating the presence of regulation on the TbTim17 levels [79,106]. Furthermore, RNAi-mediated downregulation of TbTim50 increased tolerance of the insect-dwelling form of *T. brucei* to the exogenous oxidative stress [137,138], suggesting a role of mitochondrial protein translocators in stress tolerance in this organism.

### 8.2. Connection of Tim17 Expression with Cancer

Metabolic plasticity is critical for cancer cell growth and proliferation in various tumor microenvironments [139,140]. For this purpose, mitochondrial flexibility, such as stimulation and suppression of mitochondrial activities, provides a growth advantage in many types of invasive cancer. Interestingly, a strong correlation exists between increase of hTim17A expression with adverse pathological and clinical outcomes in human breast cancer. Reduction of hTim17A expression reduced cell invasion and malignancies [141,142]. Recently, it has been shown that hTim17A expression was regulated negatively by micro-RNA, miR-133b [143]. In breast cancer cell lines and tissues, which predicted poor prognosis in breast cancer patients, miR-133b expression is inhibited by induced expression of the long non-coding RNA, NEAT1. In addition, knockdown of miR-133b or overexpression of hTim17A promotes cell migration and invasion both in vitro and in animal models, indicating that hTim17 is not only a prognostic marker, but also a potential target for cancer therapy.

## 9. Conclusions

Tim17 and its homologues have been found in all eukaryotes. Studies done primarily in yeast show that ScTim17 is an essential structural unit of the TIM23 complex. ScTim17 also is involved in dynamic remodeling of this complex, necessary for import of proteins, and acts as a gatekeeper for the TIM23 channel. However, very little is known regarding the function, protein–protein interactions, and biogenesis of Tim17 in organisms such as trypanosomatids and other members of this eukaryotic supergroup. It is also not clear how the TbTIM import channel is formed and why small TbTims are crucial for TbTim17 stability. Researchers also have a special interest in solving the mystery of how TbTim17 not only mediates import of various kinds of mitochondrial proteins, but also tRNAs that are needed for mitochondrial translation.

## Figures and Tables

**Table 1:** Different types of mitochondrial targeting signals.

Type	Destination	Examples	Reference
N-terminal cleavable ^1^	Matrix	Most of the matrix proteins ^2^	[11,18]
Stop-transfer ^3^	MIM, IMS	Cox5a, Mia40, Tim50, Yme1, Cytb2	[18,19,20]
Internal ^4^	MIM	Mitochondrial carrier proteins (SLC25) family proteins ^5^	[21,22]
Signal/tail-anchored ^6^	MOM	Tom70, Tom20, hFis1, Bak, Bax	[13,23]
Internal presequence type ^7^	MIM	BCS1, TAO ^8^	[14,15]

^1^ N-terminal presequence consists of an ampipathic α-helix, 20 to 80 amino acids (AAs) long, that is generally cleaved when entered into the matrix. However, some presequences are not cleaved. ^2^ These include metabolic enzymes, and proteins needed for mitochondrial DNA replication, transcription, translation, and others. ^3^ The N-terminal presequence followed by a hydrophobic stretch. Once the hydrophobic region reaches the TIM23 complex, translocation stops and the preprotein is laterally sorted into the membrane. ^4^ Multiple internal signals, not very well defined. ^5^ A special class of MIM proteins that possesses three repeating modules, each with 2 TMs. ^6^ A few single pass MOM proteins possess this type of signal either at the N- or C-terminus. ^7^ Some single pass MIM proteins that have N-termini exposed in the IMS possess an internal presequence-like region, which is preceded by a hydrophobic stretch. These proteins enter into the TIM23 complex as a loop structure. ^8^ In addition to an N-terminal presequence, trypanosome alternative oxidase (TAO) possesses at least one internal presequence-like region followed by a hydrophobic strecth that could be utilized as an alternate targeting signal.

**Table 2:** Components of the TIM complexes in different organisms.

Complex	Yeast/Fungi	Human	Plant	Trypanosomatids
TIM23	Tim23 Tim17 Tim50 Tim21 Mgr2	TIMM23 TIMM17 TIMM50	Tim23 Tim17 Tim50 Tim21	
PAM	Pam18 Pam16 Pam17 Tim44 mHSP70 Mge-1	DnaJC19 ^a^ DnaJC15 ^a^ Magma ^b^ Mortalin/HSPA9 ^c^	Pam18 Pam16 Tim14 Tim44 Hsp70 Mge1	Pam27 mHSP70 Mge1 Pam16 Pam18
TIM22	Tim22 Tim54 Tim18 Sdh3 Tim12	TIMM22 TIMM29 AGK	Tim22	
Small Tims	Tim9 Tim10 Tim8 Tim13 Tim12	Tim9 Tim10a Tim10b Tim8 Tim13	Tim9 Tim10 Tim8 Tim13	Tim9 Tim10 Tim8/13 Tim11 Tim12 Tim13
TbTIM ^d^				Tim17 Tim62 Tim42 Tim50 Tim54 Rhomboid I Rhomboid II ACAD Small Tims

^a^ Human DnaJC19 and DnaJC15 are functional homologues of yeast Pam18. ^b^Magma is the homologue of yeast Pam16. ^c^ Mortalin (HSPA9) is the mitochondrial Hsp70. ^d^ TBTIM complex in trypanosomatids possesses TbTim17 and several trypanosome-specific components.

**Table 3:** Number of copies of Tim17, Tim22, and Tim23 protein isoforms in different species.

Species	Tim17	Tim22	Tim23	References
*Saccharomyces cerevisiae*	1	1	1	[11,18,42]
*Candida Albicans*	1	1	1	[93]
*Homo sapien*	2	1	2	[16,94]
*Drosophila melanogaster*	3	>1	>1	[86]
Plants (*Arabidopsis thaliana*)	3	1	2	[95,96]
Euglenozoa	1	1	1	[86]
*Trypanosoma cruzi*	1			[72,91]
*Trypanosoma brucei*	1			[72,73]
*Leishmania species*	1			[91]
*Plasmodium species*	1	1	1	[93,97]
*Giardia Intestinalis*	1			[86,92]
*Acanthameoba castellanii*	1	1	1	[86]
*Trichomonas vaginalis*	4 to 5 ^a^			[34]

^a^ Belong to the Tim17/22/23 family.

## References

[1] Mehta M.M., Weinberg S.E., Chandel N.S. (2017). Mitochondrial control of immunity: Beyond ATP. Nat. Rev. Immunol..

[2] Eisner V., Picard M., Hajnóczky G. (2018). Mitochondrial dynamics in adaptive and maladaptive cellular stress responses. Nat. Cell Biol..

[3] Calvo-Rodriguez M., Bacskai B.J. (2020). Mitochondria and calcium in Alzheimer’s disease: From cell signaling to neuronal cell death. Trends Neurosci..

[4] Shoshan-Barmatz V., Gincel D. (2003). The voltage-dependent anion channel: Characterization, modulation, and role in cell life and death. Cell. Biochem. Biophys..

[5] Schleiff E., Becker T. (2011). Common ground for protein translocation: Access control for mitochondria and chloroplasts. Nat. Rev. Mol. Cell Biol..

[6] Senkler J., Senkler M., Braun H.P. (2017). Structure and function of complex I in animals and plants—A comparative view. Physiol. Plant.

[7] Eramo M.J., Lisnyak V., Formosa L.E., Ryan M.T. (2020). The ‘mitochondrial contact site and cristae organizing system’ (MICOS) in health and human disease. J. Biochem..

[8] Truscott K.N., Brandner K., Pfanner N. (2003). Mechanism of protein import into mitochondria. Curr. Biol..

[9] Hansen K.G., Herrmann J.M. (2019). Transport of Proteins into mitochondria. Protein J..

[10] Makiuchi T., Nozaki T. (2014). Highly divergent mitochondrion-related organelles in anaerobic parasitic protozoa. Biochimie.

[11] Neupert W., Herrmann J.M. (2007). Translocation of proteins into mitochondria. Annu. Rev. Biochem..

[12] Schmidt O., Pfanner N., Meisinger C. (2010). Mitochondrial protein import: From proteomics to functional mechanisms. Nat. Rev. Mol. Cell Biol..

[13] Drwesh L., Rapaport D. (2020). Biogenesis pathways of a-helical mitochondrial outer membrane proteins. Biol. Chem..

[14] Fölsch H., Guiard B., Neupert W., Stuart R.A. (1996). Internal targeting signal of the BCS1 protein: A novel mechanism of import into mitochondria. EMBO J..

[15] Hamilton V., Singha U.K., Smith J. (2014). T, Jr., Weems, E.; Chaudhuri, M. Trypanosome alternative oxidase possesses both an N-terminal and internal mitochondrial targeting signal. Eukaryot. Cell.

[16] Bauer M.F., Gempel K., Reichert A.S., Rappold G.A., Lichtner P., Gerbitz K.D., Neupert W., Brunner M., Hofmann S. (1999). Genetic and structural characterization of the human mitochondrial inner membrane translocase. J. Mol. Biol..

[17] Lister R., Hulett J.M., Lithgow T., Whelan J. (2005). Protein import into mitochondria: Origins and functions today (review). Mol. Membr. Biol..

[18] Neupert W., Brunner M. (2002). The protein import motor of mitochondria. Nat. Rev. Mol. Cell Biol..

[19] Geissler A., Krimmer T., Bömer U., Guiard B., Rassow J., Pfanner N. (2000). Membrane potential driven protein import into mitochondria. The sorting sequence of cytochrome b(2) modulates the dealtapsi-dependence of translocation of the matrix. Mol. Biol. Cell..

[20] Glaser S.M., Miller B.R., Cumsky M.G. (1990). Removal of a hydrophobic domain within the mature portion of a mitochondrial inner membrane protein causes its mislocalization to the matrix. Mol. Cell. Biol..

[21] Kang Y., Baker M.J., Liem M., Louber J., McKenzie M., Atukorala I., Ang C.S., Keerthikumar S., Mathivanan S., Stojanovski D. (2016). Tim29 is a novel subunit of the human TIM22 translocase and is involved in complex assembly and stability. Elife.

[22] Kerscher O., Holder J., Srinivasan M., Leung R.S., Jensen R.E. (1997). The Tim54p-Tim22p complex mediates insertion of proteins into the mitochondrial inner membrane. J. Cell Biol..

[23] Becker T., Pfannschmidt S., Guiard B., Stojanovski D., Milenkovic D., Kutik S., Pfanner N., Meisinger C., Wiedemann N. (2008). Biogenesis of the mitochondrial TOM complex: Mim1 promotes insertion and assembly of signal-anchored receptors. J. Biol. Chem..

[24] Becker T., Wagner R. (2018). Mitochondrial Outer Membrane Channels: Emerging Diversity in Transport Processes. Bioessays.

[25] Dekker P.J.T., Ryan M.T., Brix J., Müller H., Hönlinger A., Pfanner N. (1998). Preprotein translocase of the outer mitochondrial membrane: Molecular dissection and assembly of the general import pore complex. Mol. Cell. Biol..

[26] Künkele K.-P., Heins S., Dembowski M., Nargang F.E., Benz R., Thieffry M., Walz J., Lill R., Nussberger S., Neupert W. (1998). The preprotein translocation channel of the outer membrane of mitochondria. Cell.

[27] Araiso Y., Tsutsumi A., Qiu J., Imai K., Shiota T., Song J., Lindau C., Wenz L.S., Sakaue H., Yunoki K. (2019). Structure of the mitochondrial import gate reveals. Distinct preprotein paths. Nature.

[28] Suzuki H., Okazawa Y., Komiya T., Saeki K., Mekada E., Kitada S., Ito A., Mihara K. (2000). Characterization of rat TOM40, a central component of the preprotein translocase of the mitochondrial outer membrane. J. Biol. Chem..

[29] Ghifari A.S., Gill-Hille M., Murcha M.W. (2018). Plant mitochondrial protein import: The ins and outs. Biochem. J..

[30] Panigrahi R., Whelan J., Vrielink A. (2014). Exploring ligand recognition, selectivity and dynamics of TPR domains of chloroplast Toc64 and mitochondrial Om64 from Arabidopsis thaliana. J. Mol. Recognit..

[31] Kozjak V. (2003). Wiedemann, N.; Milenkovic, D.; Lohaus, C.; Meyer, H.E.; Guiard, B.; Meisinger, C.; Pfanner, N. An essential role of Sam50 in the protein sorting and assembly machinery of the mitochondrial outer membrane. J. Biol. Chem..

[32] Dolezal P., Dagley M.J., Kono M., Wolynec P., Likić V.A., Foo J.H., Sedinová M., Tachezy J., Bachmann A., Bruchhaus I. (2010). The essentials of protein import in the degggerate mitochondrion of Entamoeba histolytica. PLoS Pathog..

[33] Dagley M.J., Dolezal P., Likic V.A., Smid O., Purcell A.W., Buchanan S.K., Tachezy J., Lithgow T. (2009). The protein import channel in the outer mitosomal membrane of Giardia intestinalis. Mol. Biol. Evol..

[34] Rada P., Doležal P., Jedelský P.L., Bursac D., Perry A.J., Šedinová M., Smíšková K., Novotný M., Beltrán N.C., Hrdý I. (2011). The core components of organelle biogenesis and membrane transport in the hydrogenosomes of *Trichomonas vaginalis*. PLoS ONE.

[35] Makki A., Rada P., Zarsky V., Kereiche S., Kovacik L., Novotny M., Jores T., Rapaport D., Tachezy J. (2001). Triplet-pore structure of a highly divergent TOM complex of hydrogenosomes in Trichomonas vaginalis. PLoS Biol..

[36] Bomer U., Rassow J., Zufall N., Pfanner N., Meijer M., Maarse A.C. (1996). The preprotein translocase of the inner mitochondrial membrane: Evolutionary conservation of targeting and assembly of Tim17. J. Mol. Biol..

[37] Herrmann J.M., Neupert W. (2003). Protein insertion into the inner membrane of mitochondria. IUBMB Life.

[38] Schendzielorz A.B., Bragoszewski P., Naumenko N., Gomkale R., Schulz C., Guiard B., Chacinska A., Rehling P. (2018). Motor recruitment to the TIM23 channel’s lateral gate restricts polypeptide release into the inner membrane. Nat. Commun..

[39] Sirrenberg C., Bauer M.F., Guiard B., Neupert W., Brunner M. (1996). Import of carrier proteins into the mitochondria inner membrane mediated by Tim22. Nature.

[40] Koehler C.M., Jarosch E., Tokatlidis K., Schmid K., Schweyen R.J., Schatz G. (1998). Import of mitochondrial carriers mediated by essential proteins of the intermembrane space. Science.

[41] Kolli R., Soll J., Carrie C. (2018). Plant Mitochondrial Inner Membrane Protein Insertion. Int. J. Mol. Sci..

[42] Rassow J., Dekker P.J., van Wilpe S., Meijer M., Soll J. (1999). The preprotein translocase of the mitochondrial inner membrane: Function and evolution. J. Mol. Biol..

[43] Truscott K.N., Kovermann P., Geissler A., Merlin A., Meijer M., Driessen A.J., Rassow J., Pfanner N., Wagner R. (2001). A presequence- and voltage-sensitive channel of the mitochondrial preprotein translocase formed by Tim23. Nat. Struct. Biol..

[44] Rehling P., Model K., Brandner K., Kovermann P., Sickmann A., Meyer H.E., Kuhlbrandt W., Wagner R., Truscott K.N., Pfanner N. (2003). Protein insertion into the mitochondrial inner membrane by a twin-pore translocase. Science.

[45] Martinez-Caballero S., Grigoriev S.M., Herrmann J.M. (2007). Tim17p regulates the twin pore structure and voltage gating of the mitochondrial protein import complex TIM23. J. Biol. Chem..

[46] Mokranjac D., Paschen S.A., Kozany C., Prokisch H., Hoppins S.C., Nargang F.E., Neupert W., Hell K. (2003). Tim50, a novel component of the TIM23 preprotein translocase of mitochondria. EMBO J..

[47] Tamura Y., Harada Y., Shiota T., Yamano K., Watanabe K., Yokota M., Yamamoto H., Sesaki H., Endo T. (2009). Tim23-Tim50 pair coordinates functions of translocators and motor proteins in mitochondrial protein import. J. Cell Biol..

[48] Matta S.K., Kumar A., D’Silva P. (2020). Mgr2 regulates mitochondrial preprtein import by associating with channel-forming Tim23 subunit. Mol. Biol. Cell..

[49] Callegari S., Richter F., Chojnacka K., Jans D.C., Lorenzi I., Pacheu-Grau D., Jakobs S., Lenz C., Urlaub H., Dudek J. (2016). TIM29 is a subunit of the human carrier translocase required for protein transport. FEBS Lett..

[50] Kerscher O., Sepuri N.B., Jensen R.E. (2000). Tim18p is a new component of the Tim54p-Tim22p translocon in the mitochondrial inner membrane. Mol. Biol. Cell.

[51] Mårtensson C.U., Becker T. (2017). Mitochondrial protein transport meets lipid biosynthesis. Trends. Cell Biol..

[52] Qi L., Wang Q., Guan Z., Wu Y., Shen C., Hong S., Cao J., Zhang X., Yan C., Yin P. (2020). Cryo-EM structure of the human mitochondrial translocase TIM22 complex. Cell Res..

[53] Zhang Y., Ou X., Wang X., Sun D., Zhou X., Wu X., Li Q., Li L. (2020). Structure of the mitochondrial TIM22 complex from yeast. Cell Res..

[54] Bömer U., Meijer M., Maarse A.C., Hönlinger A., Dekker P.J., Pfanner N., Rassow J. (1997). Multiple interactions of components mediating preprotein translocation across the inner mitochondrial membrane. EMBO J..

[55] Ting S.Y., Schilke B.A., Hayashi M. (2014). Architecture of the TIM23 inner mitochondrial translocon and interaction with the matrix import motor. J. Biol Chem..

[56] Schilke B.A., Hayashi M., Craig E.A. (2012). Genetic analysis. Of complex interactions among. Components of the mitochondrial import motor and translocon in Saccharomyces cerevisiae. Genetics.

[57] Sinha D., Srivastava S., D’Silva P. (2016). Functional diversity of human mitochondrial J-protein is independent of their association with the inner membrane presequence translocase. J. Biol. Chem..

[58] Wiedemann N., Pfanner N., Chacinska A. (2006). Chaperoning through the mitochondrial intermembrane space. Mol. Cell.

[59] Baker M.J., Webb C.T., Stroud D.A., Palmer C.S., Frazier A.E., Guiard B., Chacinska A., Gulbis J.M., Ryan M.T. (2009). Structural and functional requirements for activity of the Tim9-Tim10. Mol. Biol. Cell.

[60] Weinhäupl K., Lindau C., Hessel A., Wang Y., Schütze C., Jores T., Melchionda L., Schönfisch B., Kalbacher H., Bersch B. (2018). Structural basis of membrane protein chaoeroning through the mitochondrial intermembrane space. Cell.

[61] Gebert N., Chacinska A., Wagner K., Guiard B., Koehler C.M., Rehling P., Pfanner N., Wiedemann N. (2008). Assembly of the three small Tim proteins precedes docking to the mitochondrial carrier translocase. EMBO Rep..

[62] Hoppins S.C., Nargang F.E. (2004). The Tim8–13 complex of Neurospora crassa functions in the assembly of proteins into both mitochondrial membranes. J. Biol. Chem..

[63] Ceh-Pavia E., Spiller M.P., Lu H. (2013). Folding and biogenesis of mitochondrial small Tim proteins. Int. J. Mol Sci..

[64] Mühlenbein N., Hofmann S., Rothbauer U., Bauer M.F. (2004). Organization and function of the small Tim complexes acting along the import pathway of metabolite carriers into mammalian mitochondria. J. Biol. Chem..

[65] Schneider A. (2018). Mitochondrial protein import in trypanosomatids: Variations on a theme or fundamentally different?. PLoS Pathog..

[66] Eckers E., Cyrklaff M., Simpson L., Deponte M. (2012). Mitochondrial protein import pathways are functionally conserved among eukaryotes despite compositional diversity of the import machineries. Biol. Chem..

[67] Pusnik M., Schmidt O., Perry A.J., Oeljeklaus S., Niemann M., Warscheid B., Lithgow T., Meisinger C., Schneider A. (2011). Mitochondrial preprotein translocase of trypanosomatids has a bacterial origin. Curr. Biol..

[68] Harsman A., Niemann M., Pusnik M., Schmidt O., Burmann B.M., Hiller S., Meisinger C., Schneider A., Wagner R. (2012). Bacterial origin of a mitochondrial outer membrane protein translocase: New paerspectives from comparative single channel electrophysiology. J. Biol. Chem..

[69] Mani J., Meisinger C., Schneider A. (2016). Peeping at TOMs-diverse entry gates to mitochondria provide insights into the evolution of Eukaryotes. Mol. Biol. Evol..

[70] Mani J., Rout S., Desy S., Schneider A. (2017). Mitochondrial protein import – Functional analysis of the highly diverged Tom22 orthologue of Trypanosoma brucei. Sci. Rep..

[71] Singha U.K., Peprah E., Williams S., Walker R., Saha L., Chaudhuri M. (2008). Characterization of the mitochondrial inner membrane protein translocator Tim17 from *Trypanosoma brucei*. Mol. Biochem. Parasitol..

[72] Singha U.K., Hamilton V., Duncan M.R., Weems E., Tripathi M.K., Chaudhuri M. (2012). Protein translocase of mitochondrial inner membrane in *Trypanosoma brucei*. J. Biol. Chem..

[73] Weems E., Singha U.K., Hamilton V., Smith J.T., Waegemann K., Mokranjac D., Chaudhuri M. (2015). Functional complementation analyses reveal that the single PRAT family protein of *Trypanosoma brucei* is a divergent homolog of Tim17 in *Saccharomyces cerevisiae*. Eukaryot. Cell.

[74] Duncan M.R., Fullerton M., Chaudhuri M. (2013). Tim50 in *Trypanosoma brucei* possesses a dual specificity phosphatase activity and is critical for mitochondrial protein import. J. Biol. Chem..

[75] Harsman A., Oeljeklaus S., Wenger C., Huot J.L., Warscheid B., Schneider A. (2016). The non-canonical mitochondrial inner membrane presequence translocase of trypanosomatids contains two essential rhomboid-like proteins. Nat. Commun..

[76] Týč J., Klingbeil M.M., Lukeš J. (2015). Mitochondrial heat shock protein machinery hsp70/hsp40 is indispensable for proper mitochondrial DNA maintenance and replication. mBio..

[77] Mensa-Wilmot K., Hoffman B., Wiedeman J., Sullenberger C., Sharma A. (2019). Kinetoplast division. Factors in a trypanosome. Trends Parasitol..

[78] Von Käne L.C., Muñoz-Gómez S.A., Oeljeklaus S., Wenger C., Warscheid B., Wideman J.G., Harsman A., Schneider A. (2020). Homologue replacement in the import motor of the mitochondrial inner membrane of trypanosomes. Elife.

[79] Singha U.K., Hamilton V., Chaudhuri M. (2015). Tim62, a Novel Mitochondrial Protein in *Trypanosoma brucei*, Is Essential for Assembly and Stability of the TbTim17 Protein Complex. J. Biol. Chem..

[80] Schneider A. (2011). Mitochondrial tRNA import and its consequences for mitochondrial translation. Annu. Rev. Biochem..

[81] Niemann M., Harsman A., Mani J., Peikert C.D., Oeljeklaus S., Warscheid B., Wagner R., Schneider A. (2017). tRNAs and proteins use the same import channel for translocation across the mitochondrial outer membrane of trypanosomes. Proc. Natl. Acad. Sci. USA.

[82] Barozai M.Y.K., Chaudhuri M. (2020). Role of the translocase of the mitochondrial inner membrane in the import of tRNAs into mitochondria in *Trypanosoma brucei*. Gene.

[83] Zikova A., Verner Z., Nenarokova A., Michels P.A.M., Lukes J. (2017). A paradigm shift: The mitoproteomes of procyclic and bloodstream *Trypanosoma brucei* are comparably complex. PLoS Pathog..

[84] Chaudhuri M., Nargang F.E. (2003). Import and assembly of Neurospora crassa Tom40 into mitochondria of *Trypanosoma brucei* in vivo. Curr. Genet..

[85] Tschopp F., Charrière F., Schneider A. (2011). In vivo study in *Trypanosoma brucei* links mitochondrial transfer RNA import to mitochondrial protein import. EMBO Rep..

[86] Žárský V., Doležal P. (2016). Evolution of the Tim17 protein family. Biol. Direct..

[87] Rossig C., Reinbothe C., Gray J., Valdes O., von Wettstein D., Reinbothe S. (2013). Three proteins mediate import of transit sequence-less precursors into the inner envelope of chloroplasts in Arabidopsis thaliana. Proc. Natl. Acad. Sci. USA.

[88] Reguenga C., Oliveira M.E., Gouveia A.M., Eckerskorn C., Sá-Miranda C., Azevedo J.E. (1999). Identification of a 24 kDa intrinsic membrane protein from mammalian peroxisomes. Biochim. Biophys. Acta.

[89] Guarani V., Paulo J., Zhai B., Huttlin E.L., Gygi S.P., Harper J.W. (2014). TIMMDC1/C3orf1 functions as a membrane-embedded mitochondrial complex I assembly factor through association with the MCIA complex. Mol. Cell. Biol..

[90] Wang Y., Carrie C., Giraud E., Elhafez D., Narsai R., Duncan O., Whelan J., Murcha M.W. (2012). location of the mitochondrial preprotein transporters B14.7 and Tim23–2 in complex I and the TIM17:23 complex in Arabidopsis links mitochondrial activity and biogenesis. Plant Cell.

[91] Gentle I.E., Perry A.J., Alcock F.H., Likić V.A., Dolezal P., Ng E.T., Purcell A.W., McConnville M., Naderer T., Chanez A.-L. (2007). Conserved motifs reveal details of ancestry and structure in the small TIM chaperones of the mitochondrial intermembrane space. Mol. Biol. Evol..

[92] Pyrihova E., Motyckova A., Voleman L., Wandyszewska N., Fiser R., Seydlova G., Roger A., Kolisko M., Dolezal P. (2018). A Single Tim Translocase in the Mitosomes of Giardia intestinalis Illustrates Convergence of Protein Import Machines in Anaerobic Eukaryotes. Genome Biol. Evol..

[93] Hewitt V.L., Heinz E., Shingu-Vazquez M., Qu Y., Jelicic B., Lo T.L., Beilharz T.H., Dumsda Y.G., Gabriel K., Traven A. (2012). A model system for mitochondrial biogenesis reveals evolutionary rewiring of protein import and membrane assembly pathways. Proc. Natl. Acad. Sci. USA.

[94] Sinha D., Srivastava S., Krishna L., D’Silva P. (2014). Unraveling the intricate organization of mammalian mitochondrial presequence translocase: Existence of multiple translocases for maintenance of mitochondrial function. Mol. Cell Biol..

[95] Murcha M.W., Lister R., Ho A.Y., Whelan J. (2003). Identification, expression, and import of components 17 and 23 of the inner mitochondrial membrane translocase from Arabidopsis. Plant Physiol..

[96] Murcha M.W., Elhafez D., Lister R., Tonti-Filippini J., Baumgartner M., Philippar K., Carrie C., Mokranjac D., Soll J., Whelan J. (2007). Characterization of the preprotein and amino acid transporter gene family in Arabidopsis. Plant Physiol..

[97] Heinz E., Lithgow T. (2013). Back to basics: A revealing secondary reduction of the mitochondrial protein import pathway in diverse intracellular parasites. Biochim. Biophys. Acta.

[98] Maarse A.C., Blom J., Keil P., Pfanner N., Meijer M. (1994). Identification of the essential yeast protein MIM17, an integral mitochondrial inner membrane protein involved in protein import. FEBS Lett..

[99] Kübrich M., Keil P., Rassow J., Dekker P.J., Blom J., Meijer M., Pfanner N. (1994). The polytopic mitochondrial inner membrane protein MIM17 and MIM23 operates at the same preprotein import site. FEBS Lett..

[100] Berthold J., Bauer M.F., Schneider H.C., Klaus C., Dietmeier K., Neupert W., Brunner M. (1995). The MIM complex mediates preprotein translocation across the mitochondrial inner membrane and couples it to the mt-Hsp70/ATP driving system. Cell.

[101] Dekker P.J., Martin F., Maarse A.C., Bömer U., Müller H., Guiard B., Meijer M., Rassow J., Pfanner N. (1997). The Tim core complex defines the number of mitochondrial translocation contact sites and can hold arrested preproteins in the absence of matrix Hsp70-Tim44. EMBO J..

[102] Van der Laan M., Hutu D.P., Rehling P. (2010). On the mechanism of preprotein import by the mitochondrial presequence translocase. Biochim. Biophys. Acta.

[103] Chacinska A., Lind M., Frazier A.E., Dudek J., Meisinger C., Geissler A., Sickmann A., Meyer H.E., Truscott K.N., Guiard B. (2005). Mitochondrial presequence translocase: Switching between TOM tethering and motor recruitement involves Tim21 and Tim17. Cell.

[104] Singha U.K., Tripathi A., Smith J.T., Quinones L., Saha A., Singha T., Chaudhuri M. (2020). Tim54, a novel IM-associated protein, is involved in the import of the internal signal-containing mitochondrial protein in *Trypanosoma brucei*. Biol. Cell.

[105] Wenger C., Oeljeklaus S., Warscheid B., Schneider A., Harsman A. (2017). A trypanosomal orthologue of an intermembrane space chaperone has a non-canonical function in biogenesis of the single mitochondrial inner membrane protein translocase. PLoS Pathog..

[106] Smith J.T., Singha U.K., Misra S., Chaudhuri M. (2018). Divergent Small Tim Homologues Are Associated with TbTim17 and Critical for the Biogenesis of TbTim17 Protein Complexes in *Trypanosoma brucei*. mSphere.

[107] Donzeau M., Káldi K., Adam A., Paschen S., Wanner G., Guiard B., Bauer M.F., Neupert W., Brunner M. (2000). links the inner and outer mitochondrial membrane. Cell.

[108] Bauer M.F., Sirrenberg C., Neupert W., Brunner M. (1996). Role of Tim23 as voltage sensor and presequence receptor in protein import into mitochondria. Cell.

[109] Geissler A., Chacinska A., Truscott K.N., Wiedemann N., Brandner K., Sickmann A., Meyer H.E., Meisinger C., Pfanner N., Rehling P. (2002). The mitochondrial presequence translocase: An essential role of Tim50 in directing preproteins to the import channel. Cell.

[110] Waegemann K., Popov-Čeleketić D., Neupert W., Azem A., Mokranjac D. (2015). Cooperation of TOM and TIM23 complexes during translocation of proteins into mitochondria. J. Mol. Biol..

[111] Popov-Celeketić D., Mapa K., Neupert W., Mokranjac D. (2008). Active remodeling of the TIM23 complex during translocation of preproteins into mitochondria. EMBO J..

[112] Günsel U., Paz E., Gupta R., Mathes I., Azem A., Mokranjac D. (2020). In vivo dissection of the Intrinsically disordered receptor domain of Tim23. J. Mol. Biol..

[113] Káldi K., Bauer M.F., Sirrenberg C., Neupert W., Brunner M. (1998). Biogenesis of Tim23 and Tim17, integral components of the TIM machinery for matrix-targeted preproteins. EMBO J..

[114] Rothbauer U., Hofmann S., Mühlenbein N., Paschen S.A., Gerbitz K.D., Neupert W., Brunner M., Bauer M.F. (2001). Role of the deafness dystonia peptide 1 (DDP1) in import of humanTim23 into the inner membrane of mitochondria. J. Biol. Chem..

[115] Davis A.J., Alder N.N., Jensen R.E., Johnson A.E. (2007). The Tim9p/Tim10p and Tim8p/Tim13p complexes bind to specific sites on Tim23 during mitochondrial protein import. Mol. Biol. Cell.

[116] Beverly K.N., Sawaya M.R., Schmid E., Koehler C.M. (2008). The Tim8-Tim13 complex has multiple substrate binding sites and binds cooperatively to Tim23. J. Mol. Biol..

[117] Meier S., Neupert W., Herrmann J.M. (2005). Conserved N-terminal negative charges in the Tim17 subunit of the TIM23 translocase play a critical role in the import of preproteins into mitochondria. J. Biol. Chem..

[118] Ramesh A., Peleh V., Martinez-Caballero S., Wollweber F., Sommer F., van der Laan M., Schroda M., Alexander R.T., Campo M.L., Herrmann J.M. (2016). A disulfide bond in the TIM23 complex is crucial for voltage gating and mitochondrial protein import. J. Cell Biol..

[119] Wrobel L., Sokol A.M., Chojnacka M., Chacinska A. (2016). The presence of disulfide bonds reveals an evolutionary conserved mechanism involved in mitochondrial protein translocase assembly. Sci. Rep..

[120] Weems E., Singha U.K., Smith J.T., Chaudhuri M. (2017). The divergent N-terminal domain of Tim17 is critical for its assembly in the TIM complex in *Trypanosoma brucei*. Mol. Biochem. Parasitol..

[121] Matta S.K., Pareek G., Bankapalli K., Oblesha A., D’Silva P. (2017). Role of Tim17 transmembrane regions in regulating the Architecture of presequence translocase and mitochondrial DNA stability. Mol. Cell. Biol..

[122] Demishtein-Zohary K., Günsel U., Marom M., Banerjee R., Neupert W., Azem A., Mokranjac D. (2017). Role of Tim17 in coupling the import motor to the translocation channel. Of the mitochondrial presequence translocase. Elife.

[123] Murcha M.W., Wang Y., Whelan J. (2012). A molecular link between mitochondrial preprotein transporters and respiratory chain complex. Plant Signal Behav..

[124] Mokranjac D., Popov-Celeketić D., Hell K., Neupert W. (2005). Role of Tim21 in mitochondrial translocation contact sites. J. Biol. Chem..

[125] van der Laan M., Wiedemann N., Mick D.U., Guiard B., Rehling P., Pfanner N. (2006). A role for Tim21 in membrane-potential-dependent preporting sorting in mitochondria. Curr. Biol..

[126] Rainbolt T.K., Atanassova N., Genereux J.C., Wiseman R.L. (2013). Stress-regulated translational attenuation adapts mitochondrial protein import through Tim17A degradation. Cell Metab..

[127] Schmidt O., Harbauer A.B., Rao S., Eyrich B., Zahedi R.P., Stojanovski D., Schönfisch B., Guiard B., Sickmann A., Pfanner N. (2011). Regulation of mitochondrial protein import by cytosolic kinases. Cell.

[128] Rao S., Schmidt O., Harbauer A.B., Schönfisch B., Guiard B., Pfanner N., Meisinger C. (2012). Biogenesis of the preprotein translocase of the outer mitochondrial membrane: Protein kinase A phosphorylates the precursor Tom40 and impairs its import. Mol. Biol. Cell..

[129] Pellegrino M.W., Nargund A.M., Haynes C.M. (2013). Signaling the mitochondrial unfolded protein response. Biochim. Biophys. Acta Mol. Cell. Biol. Lipids.

[130] Tran H.C., Van Aken O. (2020). Mitochondrial unfolded protein-related response across kingdoms: Similar problems, different regulators. Mitochondrion.

[131] Nargund A.M., Pellegrino M.W., Fiorese C.J., Baker B.M., Haynes C.M. (2012). Mitochondrial import efficiency of ATFS-1 regulates mitochondrial UPR activation. Science.

[132] Nargund A.M., Fiorese C.J., Pellegrino M.W., Deng P., Haynes C.M. (2015). Mitochondrial and nuclear accumulation of the transcription factor ATFS-1 promotes OXPHOS recovery during the UPR(mt). Mol. Cell..

[133] Zöller E., Laborenz J., Krämer L., Boos F., Räschle M., Alexander R.T., Herrmann J.M. (2020). The intermembrane space protein Mix23 is a novel stress-induced mitochondrial import factor. J. Biol. Chem..

[134] Marada A., Allu P.K., Murari A., PullaReddy B., Tammineni P., Thiriveedi V.R., Danduprolu J., Sepuri N.B. (2013). Mge1, a nucleotide exchange factor of Hsp70 acts as an oxidative sensor to regulate mitochondrial Hsp70 function. Mol. Biol. Cell.

[135] Srivastava S., Sinha D., Saha P.P., Marthala H., D’Silva P. (2014). Maggmas functions as a ROS reglator and provides cytoprotection against oxidative stress-mediated damage. Cell Death Dis..

[136] Wojityla L., Kosmala A., Garnczarska M. (2013). Lupine embryo axes under salinity stress. II. Mitochondrial proteome response. Acta Physiol. Plant.

[137] Fullerton M., Singha U.K., Duncan M., Chaudhuri M. (2015). Down regulation of Tim50 in *Trypanosoma brucei* increases tolerance to oxidative stress. Mol. Biochem. Parasitol..

[138] Tripathi A., Singh U.K., Paromov V., Hill S., Pratap S., Rose K., Chaudhuri M. (2019). The crosstalk between TbTim50 and PIP39, two aspartate-based protein phosphatases, maintain cellular homeostasis in *Trypanosoma brucei*. mSphere.

[139] Avagliano A., Ruocco M.R., Aliotta F., Belviso I., Accurso A., Masone S., Montagnani S., Arcucci A. (2019). Mitochondrial Flexibility of Breast Cancers: A growth advantage and a therapeutic opportunity. Cell.

[140] Fulda S., Galluzzi L., Kroemer G. (2010). Targeting mitochondria for cancer therapy. Nat. Rev. Drug Discov..

[141] Yang X., Si Y., Tao T., Martin T.A., Cheng S., Yu H., Li J., He J., Jiang W.G. (2016). The Impact of TIMM17A on Aggressiveness of human breast cancer cells. Anticancer Res..

[142] Salhab M., Patani N., Jiang W., Mokbel K. (2012). High TIMM17A expression is associated with adverse pathological and clinical outcomes in human breast cancer. Breast Cancer.

[143] Li X., Deng S., Pang X., Song Y., Luo S., Jin L., Pan Y. (2019). LncRNA NEAT1 silenced miR-133b promotes migration and invasion of breast cancer cells. Int. J. Mol. Sci..

